# Bovine Dialyzable Leukocyte Extract IMMUNEPOTENT-CRP Induces Selective ROS-Dependent Apoptosis in T-Acute Lymphoblastic Leukemia Cell Lines

**DOI:** 10.1155/2020/1598503

**Published:** 2020-06-08

**Authors:** Helen Yarimet Lorenzo-Anota, Ana Carolina Martínez-Torres, Daniel Scott-Algara, Reyes S. Tamez-Guerra, Cristina Rodríguez-Padilla

**Affiliations:** ^1^Universidad Autónoma de Nuevo León, Facultad de Ciencias Biologicas, Laboratorio de Inmunología y Virología, San Nicolás De Los Garza, Mexico; ^2^Unité de Biologie Cellulaire des Lymphocytes, Institut Pasteur, Paris, France; ^3^Longeveden, SA de CV, Monterrey, Mexico

## Abstract

Immunotherapies strengthen the immune system to fight multiple diseases such as infections, immunodeficiencies, and autoimmune diseases, and recently, they are being used as an adjuvant in cancer treatment. IMMUNEPOTENT-CRP (I-CRP) is an immunotherapy made of bovine dialyzable leukocyte extract (bDLE) that has chemoprotective and immunomodulatory effects in different cellular populations of the immune system and antitumor activity in different cancer cell lines. Our recent results suggest that the antineoplastic effect of I-CRP is due to the characteristics of cancer cells. To confirm, we evaluated whether the selectivity is due to cell lineage or characteristics of cancer cells, testing cytotoxicity in T-acute lymphoblastic leukemia cells and their cell death mechanism. Here, we assessed the effect of I-CRP on cell viability and cell death. To determine the mechanism of cell death, we tested cell cycle, mitochondrial and nuclear alterations, and caspases and reactive oxygen species (ROS) and their role in cell death mechanism. Our results show that I-CRP does not affect cell viability in noncancer cells and induces selective cytotoxicity in a dose-dependent manner in leukemic cell lines. I-CRP also induces mitochondrial damage through proapoptotic and antiapoptotic protein modulation (Bax and Bcl-2) and ROS production, nuclear alterations including DNA damage (*γ*-H2Ax), overexpression of p53, cell cycle arrest, and DNA degradation. I-CRP induced ROS-dependent apoptosis in leukemic cells. Overall, here, we show that I-CRP cytotoxicity is selective to leukemic cells, inducing ROS-dependent apoptosis. This research opens the door to further exploration of their role in the immune system and the cell death mechanism that could potentially work in conjunction with other therapies including hematological malignances.

## 1. Introduction

Regulated cell death (RCD) is a mechanism by which the cell activates its own machinery for self-destruction; it involves structured signaling cascades and molecularly defined effector mechanisms and is important for the maintenance of tissues [1–4]. Apoptosis is the most widely described RCD mechanism; it is characterized by cell shrinkage (pyknosis), membrane blebbing, apoptotic body formation, DNA fragmentation (karyorrhexis), and chromatin condensation [5–7]. In cancer, there is a break in the circuits that regulate division and regulated cell death, leading to deregulation of apoptosis and generating uncontrolled cell growth [1].

T-cell acute lymphoblastic leukemia (T-ALL) is a type of cancer derived from the bone marrow that affects T-lymphocytes and is the most common cause of cancer in children worldwide [8, 9]. Malignant progression is promoted by the myeloprotection provided by the bone marrow and evasion of the host's immune system, evidencing the resistance capacity to treatments of the leukemic cells [8, 10]. At present, the main therapies for leukemia include combinations of chemotherapies and corticosteroids, kinase inhibitors, and stem cell transplant [8, 11]; however, they cause important side effects in healthy immune system cells. Therefore, it is important to develop therapies that eliminate cancer cells without affecting healthy immune system cells and stimulate the immune system to fight cancer.

Immunotherapy consists of treatments that use the immune system to fight multiple diseases such as infections, autoimmune diseases, immunodeficiencies, and lately, in cancer treatment as an adjuvant [12, 13]. IMMUNEPOTENT-CRP© (I-CRP) is an immunotherapeutic agent composed of bovine dialyzable leukocyte extract (bDLE) obtained from disintegrated spleen. In previous reports, I-CRP showed a potential immunomodulatory effect in preclinical trials of breast cancer treatments, [14, 15] and in *in vivo* assays, it showed an antitumor effect [16, 17]. Several *in vitro* studies reveal its immunomodulatory properties in human and mouse monocytes and macrophages [18, 19] and their cytotoxic effect in different cancer cell lines [20, 21]. In the breast cancer cell line MCF-7, I-CRP inhibits cell growth, suppresses DNA-binding activity of AP-1, decreases c-Jun protein expression, and modulates the mRNA expression of cell death proteins such as NF*κ*B, p53, c-myc, bax, and bcl-2 [22]. Also, in the cervical cancer cell line HeLa, I-CRP decreases cell viability through cell cycle arrest in the G2/M phase with caspase-3 activation and ROS production, inducing caspase-independent but ROS-dependent RCD [21]. In lung cancer cells, it induces cell cycle arrest, cell death, and also caspase-independent but ROS-dependent RCD [23].

At present, all results suggest a dual role of I-CRP: in healthy immune system cells as an immunomodulator, and in cancer cells as a cytotoxic agent. However, the effect of I-CRP on cancer derived from the immune system is unknown. The limited information of its molecular action mechanisms has hindered its widespread use in different pathologies. The aim of this research was to analyze the effect of I-CRP in T-ALL cells (human cancer cells derived from immune system) and their mechanisms of cytotoxicity. We used Molt-4 and CEM cells as T-ALL model cancer cells and assessed the mechanism of cell death by evaluating mitochondrial (ratio Bax-Bcl-2, ROS and TMRE) and nuclear (*γ*-H2Ax, cell cycle, and DNA degradation) alterations and the role of ROS and caspases in cell death mechanism.

## 2. Materials and Methods

### 2.1. Cell Culture

Molt-4 (ATCC CRL-1582) and CEM (ATCC® CCL-119™) cell lines (T-acute lymphoblastic leukemia cells) were obtained from the American Type Culture Collection (ATCC, Manassas, VA, USA) and maintained under suggested conditions. Cells were cultured in plastic sterile flasks (Corning Inc. Costar®, USA) at 37°C in 5% CO_2_ atmosphere, using the RPMI 1640 medium (GIBCO Thermo Fisher, Waltham, Massachusetts, USA) supplemented with 1 *μ*g/mL amphotericin B, 1 *μ*g/mL penicillin, 2.5 × 10^−3^ *μ*g/mL streptomycin, and 10% of FBS (GIBCO Thermo Fisher, Waltham, Massachusetts, USA).

Peripheral blood mononuclear cells (PBMC) were isolated from healthy donors after obtaining written informed consent by density gradient centrifugation with Ficoll-Paque™ PLUS (GE Healthcare, Chicago, Illinois, USA) and maintained at 4 × 10^6^ cells/mL in cell culture plates at 37°C in 5% CO_2_ atmosphere, using the RPMI 1640 medium (GIBCO Thermo Fisher, Waltham, Massachusetts, USA) supplemented with 1 *μ*g/mL amphotericin B, 1 *μ*g/mL penicillin, 2.5 × 10^−3^ *μ*g/mL streptomycin, and 10% of FBS (GIBCO Thermo Fisher, Waltham, Massachusetts, USA). This study was approved by the Institutional Ethics Committee of the College of Biological Sciences, Universidad Autónoma de Nuevo León. To analyze T-lymphocytes in PBMC culture, we used the anti-CD3 antibodies CD3*ε*-APC (BD Biosciences, San Jose, CA; cat: 555335, clone: UCHT 1) and CD3*ε*-FITC (BD Biosciences; cat: 555916, clone: UCHT 1).

### 2.2. T-Lymphocyte Purification

T-lymphocytes (CD3+) were isolated from the PBMC culture by negative selection using magnetic-activated cell sorting (MACS) microbead technology (Miltenyi Biotec, Bergisch Gladbach, Germany; >90% purity and >90% viability), with anti-CD20 microbeads (cat: 130-091-104; Miltenyi Biotec), anti-CD16 microbeads (cat: 130-045-701; Miltenyi Biotec), and anti-CD14 microbeads (cat: 130-050-20; Miltenyi Biotec) cocktail, as stated by the manufacturer's instructions. T-lymphocyte culture was maintained at same conditions of the PBMC culture.

### 2.3. IMMUNEPOTENT-CRP

The bovine dialyzable leukocyte extract IMMUNEPOTENT-CRP (I-CRP) was produced by the Immunology and Virology Laboratory of the College of Biological Sciences, Universidad Autónoma de Nuevo León (San Nicolás de los Garza, Nuevo León, México) and was dissolved in the RPMI 1640 medium (GIBCO Thermo Fisher, Waltham, Massachusetts, USA) supplemented with 1 *μ*g/mL amphotericin B, 1 *μ*g/mL penicillin, 2.5 × 10^−3^ *μ*g/mL streptomycin, and 10% of FBS (GIBCO Thermo Fisher, Waltham, Massachusetts, USA). One unit of I-CRP is defined as the product obtained from 1 × 10^8^ leukocytes.

### 2.4. Cell Viability Assessment

Cell viability was determined using calcein-AM (Thermo Fisher, Waltham, Massachusetts, USA). Briefly, cells were seeded at 5 × 10^4^ cells per well in 96-well plates (Corning Inc. Costar®, NY, USA) and exposed to different concentrations of I-CRP (0.4, 0.6, 0.8, and 1.0 U/mL; after 24 and 48 hours of treatment, the cells were washed using phosphate-buffered saline (PBS) and then resuspended in 100 *μ*L of FACS buffer and calcein-AM (2 *μ*M) for 30 minutes at room temperature; finally, the cells were washed using PBS. The cells were then assessed by flow cytometry (Fluorescence-Activated Cell Sorting (FACS); BDAccury6; Becton Dickinson, San Jose, CA, USA) and analyzed using the FlowJo software (Tree Star Inc., Ashland, OR, USA).

### 2.5. Cell Death Analysis

Cell death was determined after 24 and 48 hours of I-CRP exposure, analyzing phosphatidylserine exposure using annexin V-allophycocyanin (APC) (AnnV, 0.25 *μ*g/mL; BD Biosciences Pharmingen, San Jose, CA, USA) and cell membrane permeability with propidium iodide (PI; 0.5 *μ*g/mL; MilliporeSigma, Eugene, OR, USA) stain. Cells were seeded at 5 × 10^4^ cells per well in 96-well plates (Corning Inc. Costar®, NY, USA) and exposed to different concentrations of I-CRP (0.4, 0.6, 0.8, and 1.0 U/mL) in subsequent assays; this allowed to define the median cytotoxic concentration of I-CRP required to induce cell death by 50% (CC_50_). After treatment, cells were recollected and washed with phosphate-buffered saline (PBS), then resuspended in binding buffer (10 mM HEPES/NaOH pH 7.4, 140 mM NaCl, 2.5 mM CaCl_2_), and stained during 30 minutes at 4°C.

### 2.6. Mitochondrial Membrane Potential Assessment

To determine mitochondrial damage, we tested loss of mitochondrial membrane potential by tetramethylrhodamine ethyl ester stain (TMRE, 50 nM; Sigma, Aldrich, Darmstadt, Germany) [25]. Cells were seeded at 5 × 10^4^ cells per well in 96-well plates (Corning Inc. Costar®, NY, USA) and treated with I-CRP (CC_50_) and incubated 24 hours. After treatment, cells were recollected and washed with PBS and finally stained for 30 minutes at 37°C.

### 2.7. Western Blot Analysis

Cells treated with CC_50_ of I-CRP for 24 hours and were lysed in lysis buffer (50 mM Tris (pH 8.0), 5 mM EDTA, 150 mM NaCl, 0.5% Nonidet P-40, and 1 mM phenylmethylsulfonyl fluoride (PMSF)). Samples containing 100 *μ*g of whole cell protein were separated on 15% SDS-PAGE gels and blotted onto nitrocellulose transfer membranes. After blocking with 5% non-fat milk, each membrane was incubated overnight with a primary antibody (Santa Cruz Biotechnology, Dallas, TX, USA) against p53 (1 : 1,000), Bax (1 : 1,000), Bcl-2 (1 : 1,000), and *β*-actin (1 : 1,000), and then with a secondary anti-mouse antibody (1 : 10000) (Santa Cruz Biotechnology). Protein expression was visualized with an enhanced chemoluminescence (ECL) detection kit (Thermo Scientific, Waltham, MA, USA).

### 2.8. Reactive Oxygen Species Production Analysis

Reactive oxygen species (ROS) production levels were measured with two different stains dihydroethidium (DHE; Invitrogen, St Louis, MO, USA) and dichlorodihydrofluorescein diacetate (DCFDA; Invitrogen, St Louis, MO, USA). After treatment for 24 hours with CC_50_ of I-CRP, cells were washed with PB, stained with DHE (10 *μ*M) or DCFDA (1*μ*Μ), and incubated for 30 minutes at 37°C.

### 2.9. Nuclear Alteration Analysis

To measure DNA damage, we tested *γ*-H2AX histone phosphorylated. Cells were seeded at 5 × 10^5^ cells/mL in 6-well sterile plates (Corning Inc. Costar®, NY, USA) and treated for 24 hours with CC_50_ of I-CRP. Cells were washed with PBS and fixed by bubbling with methanol (100%) and conserved overnight at −20°C. After fixed, cells were blocked (FACS buffer 10%) for 30 minutes at 4°C and washed (FACS buffer 2%). Then, cells were incubated with a specific primary antibody to the variant phosphorylated histone (*γ*-H2AX; ABCAM, Cambridge, UK) in a 1 : 100 dilution [24] for one hour at room temperature in constant agitation. Negative controls (isotype) were incubated only with 2% FACS buffer. Then, we incubated the secondary antibody (Anti-Mouse FITC; ABCAM, Cambridge, UK) in a 1 : 100 dilution for 30 minutes at room temperature and conserved overnight at 4°C.

The p53 expression was analyzed using a specific primary antibody to p53 (p53; ABCAM, Cambridge, UK) in a 1 : 100 dilution by flow cytometry as described previously.

### 2.10. Cell Cycle Analysis

For cell cycle analysis, we quantified intracellular DNA using propidium iodide (PI) staining by flow cytometry [25]. After treatment for 24 hours with CC_50_ of I-CRP, cells were recollected and washed with PBS, and then we fixed in 70% ethanol by bubbling and conserved at −20°C overnight. Additionally, cells were washed again with PBS and incubated with RNase (MilliporeSigma, Eugene, OR, USA) and PI (10 *μ*g/mL; MilliporeSigma, Eugene, OR, USA) at 37°C for 30 minutes. For DNA degradation, we quantified the SubG1 population.

### 2.11. Cleaved Caspase-3 Analysis

We used a specific detection kit, FITC-DEV-FMK (ABCAM; Cambridge, UK) [21] to assess caspase-3 activation. In brief, the cells were seeded at 1 × 10^5^ cells per well in 96-well plates (Corning Inc. Costar®, NY, USA), and after 24 hours of treatment with I-CRP (CC_50_), they were recovered and stained following the manufacturer's instructions.

### 2.12. Pharmacological Inhibition

We used different pharmacological inhibitors to determine the cell death mechanism of I-CRP in leukemic cells. QVD-OPh (QVD, 10 *μ*M) as a general caspase inhibitor; Z-DEVD (1 *μ*M) as caspase-3 inhibitor; Z-IETD (1 *μ*M) as caspase-8 inhibitor; and Z-LEHD (1 *μ*M) as caspase-9 inhibitor were acquired from BioVision (CA, USA). N-acetyl-cysteine (NAC, 5 mM) was used as an ROS inhibitor. The inhibitors were added 30 minutes before CC_50_ I-CRP treatment. All stock solutions were wrapped in foil and stored at −20°C.

### 2.13. Statistical Analysis

Western blot analysis was measured using the ImageJ software. Statistical analyses were done using the paired Student's *t*-test. The statistical significance was defined as *p* < 0.005. The data were analyzed using GraphPad Prism (GraphPad Software, San Diego, CA, USA). The results given in this study represent the mean of at least three independent experiments done in triplicate (mean ± SD).

## 3. Results

### 3.1. IMMUNEPOTENT-CRP Decreases Selective Cell Viability in Leukemic Cells

**Figure 1:**
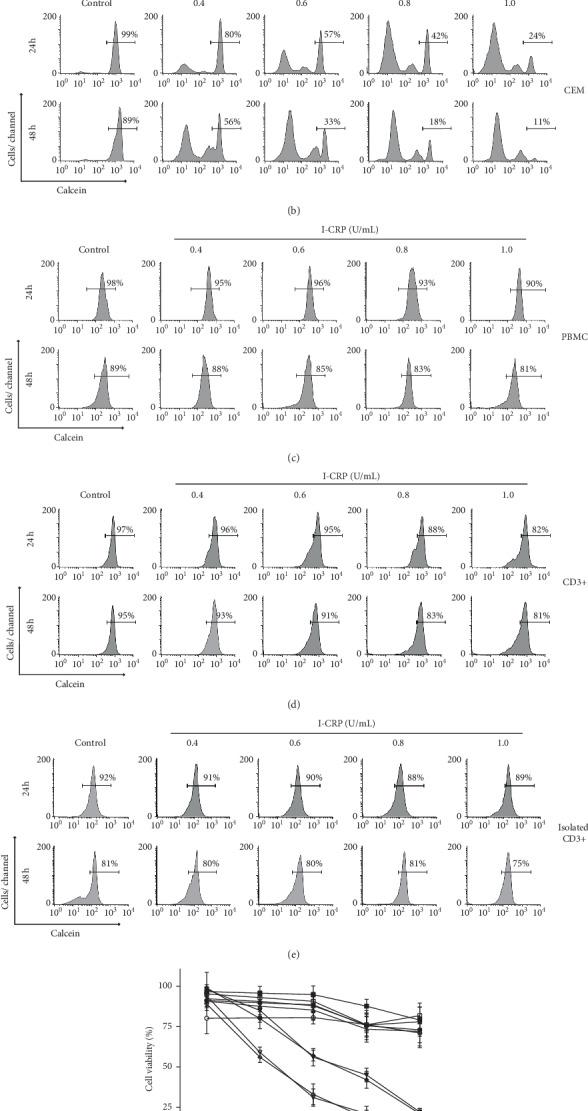
Cell viability of T-ALL cell lines and healthy counterpart after I-CRP treatment. Representative histograms of cell viability analysis by flow cytometry using calcein-AM staining in (a) Molt-4, (b) CEM, (c) PBMC, (d) CD3+ cells in PBMC, and (e) isolated CD3+ treated with different concentrations (0.4, 0.6, 0.8, and 1.0 U/mL) of I-CRP for 24 and 48 hours. (f) Quantification of cell viability. The results are presented as mean ± standard deviation of three different experiments.

We assessed whether I-CRP induces selective cytotoxicity in leukemic cells. For this, we analyzed cell viability in the T-acute lymphoblastic leukemia (T-ALL) cell lines Molt-4 and CEM and in the healthy counterpart peripheral blood mononuclear cells (PBMC) and T-lymphocytes (Figure 1). In Figure 1, we show histograms of cell viability analysis in Molt-4 (Figure 1(a)), CEM (Figure 1(b)), PBMC (Figure 1(c)), T-lymphocytes in total PBMC (CD3+) (Figure 1(d)), and in isolated T-lymphocytes (Figure 1(e)) at different concentrations of I-CRP (0.4, 0.6, 0.8, and 1.0 U/mL) at 24 and 48 hours of treatment. In Figure 1(f), we observed that I-CRP decreases cell viability in a time- and concentration-dependent manner in T-ALL cell lines; however, we observed that cell viability of the healthy counterpart was not affected, including T-lymphocytes (CD3+). These results showed that I-CRP decreases selectively the viability in malignant cells only.

### 3.2. IMMUNEPOTENT-CRP Induces Selective Cell Death in Leukemic Cell Lines

**Figure 2:**
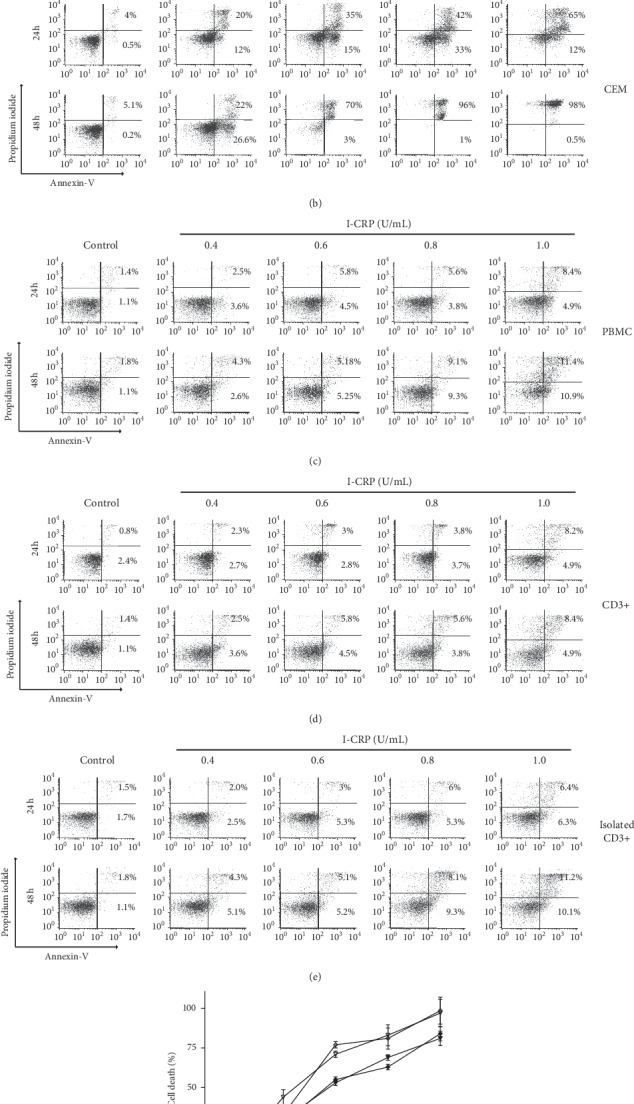
Phosphatidylserine exposure and membrane permeability of T-ALL cell lines and healthy counterpart after I-CRP treatment. Representative dot plots of cell death analysis by flow cytometry using annexin-V and propidium iodide (PI) staining in (a) Molt-4, (b) CEM, (c) PBMC, (d) CD3+ cells in PBMC, and (e) isolated CD3+ treated at different concentrations (0.4, 0.6, 0.8, and 1.0 U/mL) of I-CRP for 24 and 48 hours. (f) Quantification of cell death. The results are presented as mean ± standard deviation of three different experiments.

To confirm that the loss of cell viability is due to the cytotoxic effect of I-CRP and not due to a metabolic effect, we used a cell death assay analyzing phosphatidylserine (PS) exposure (annexin-V) and membrane permeabilization (propidium iodide, PI) at different concentrations of I-CRP (0.4, 0.6, 0.8, and 1.0 U/mL), after 24 and 48 hours of treatment (Figure 2) in T-ALL cells and the healthy counterpart. As shown in Figures 2(a) and 2(b), I-CRP at 0.8 U/mL increases cell death with double positive population for annexin-V and PI staining and enhances concentration- and time-dependent cell death at 24 and 48 hours in Molt-4 (Figure 2(a)) and CEM (Figure 2(b)) cells, and the mean cytotoxic concentration that killed 50% of the cells (CC_50_) was 0.6 U/mL at 24 hours and the CC_100_ (killed 100% of cells) was 1.0 U/mL. We did not observe affectations in the cell integrity of the healthy counterpart (Figures 2(c)–2(e)) even after 48 hours of treatment. Cell death induction by I-CRP in T-ALL cells was confirmed by microscopy assessment, where we observed morphological alterations, including apoptotic bodies and cell shrinkage (Supplementary 1). These results indicated that I-CRP is a selective cell death inductor in T-ALL cells, which does not affect healthy cells at same doses and times of treatment, thus confirming the selective cytotoxic effect in cancer cells.

### 3.3. IMMUNEPOTENT-CRP Induces Mitochondrial Alterations in Leukemic Cells

**Figure 3:**
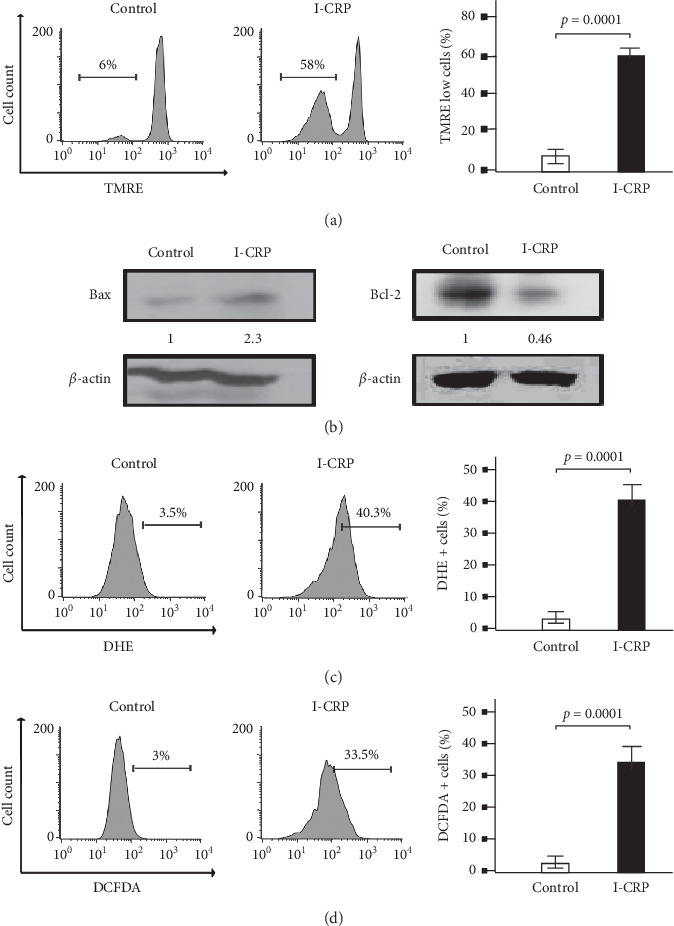
Mitochondrial alterations in Molt-4 cell line upon I-CRP. (a) Representative histogram of mitochondrial membrane potential loss analysis and quantification, using TMRE by flow cytometry. (b) Western blot analysis showed the expression of Bax and Bcl-2 proteins. The same blot was reproved with *β*-actin to confirm equal loading of each lane. (c) Representative histogram of ROS analysis and quantification by flow cytometry using DHE stain. (d) Representative histogram of ROS analysis and quantification by flow cytometry using DCFDA stain. The results are presented as mean ± standard deviation of three different experiments.

Mitochondria are the bioenergetic center of the cell and a reservoir of prodeath factors and have an essential role in the intrinsic pathway of apoptosis, activating caspase proteases [4, 26]. To elucidate the cell death mechanism induced by I-CRP in T-ALL cells, we analyzed mitochondrial alterations in Molt-4 (Figure 3) and CEM (Supplementary 2(a) and 2(b)). We tested loss of mitochondrial membrane potential (LMMP) with TMRE marker, Bax-Bcl-2 ratio (pro- and anti-apoptotic proteins) by western blot, and reactive oxygen species (ROS) production using DHE and DCFDA staining. We found that I-CRP enhances LMMP in Molt-4 from 6% to 58% (Figure 3(a)), indicating that I-CRP induces mitochondrial impairment. In Bax-Bcl-2 ratio analysis (Figure 3(b)), we showed that treatment increases Bax expression and reduces Bcl-2 expression. We also found that I-CRP increases ROS production in Molt-4 cells (Figures 3(c) and 3(d)). We did not observe mitochondrial alterations in PBMC (Supplementary 2(c) and 2(d)). These mitochondrial alterations suggest cell death by apoptosis in T-ALL cell lines.

### 3.4. IMMUNEPOTENT-CRP Induces Nuclear Alterations

**Figure 4:**
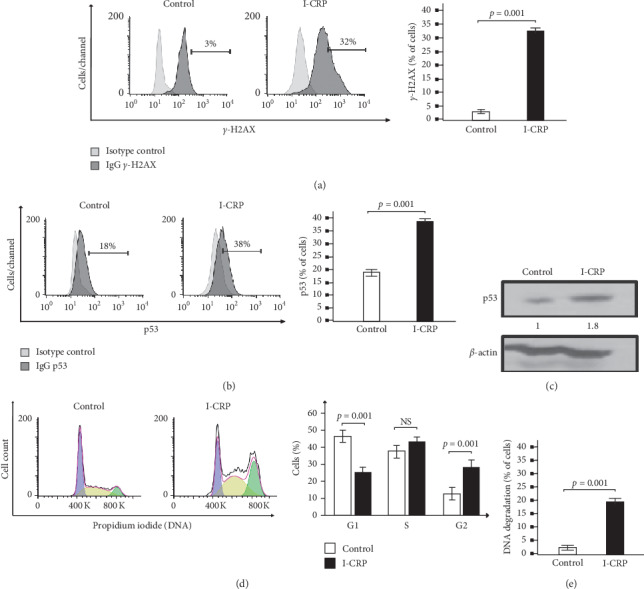
Nuclear alterations in Molt-4 cell line after I-CRP treatment. (a) Representative histogram of nuclear damage analysis and quantification measure *γ*-H2AX by flow cytometry. (b) Representative histogram of p53 analysis and quantification by flow cytometry in Molt-4 cells. (c) Western blot analysis showed the expression of p53 protein and *β*-actin to confirm equal loading of each lane. (d) Representative histogram of cell cycle analysis and quantification, using RNase and propidium iodide (PI) stain, by flow cytometry. (e) DNA degradation analysis by flow cytometry. The results are presented as mean ± standard deviation of three different experiments.

Usually RCD is associated with DNA damage. During DNA damage, H2AX, a variant of the H2A protein family, is phosphorylated in serine 139 (*γ*-H2Ax) to recruit repair proteins that can induce p53 activation, leading to cell cycle arrest followed by cell death characterized by DNA degradation [1, 4]. We tested phosphorylation of H2Ax (*γ*-H2Ax) in Molt-4 cells; we found that the percentage of *γ*-H2Ax increases from 3% to 32% in treated cells (Figure 4(a)), indicating that I-CRP induces DNA damage. Then, we analyzed p53 expression and found it increased from 18% to 38% in treated cells (Figure 4(b)). The western blot analysis (Figure 4(c)) confirmed overexpression of p53 in treated cells with I-CRP. These results suggest that I-CRP has a role in cell cycle arrest and cell death. Finally, to determine cell cycle alterations, we quantified DNA using PI staining and RNase. The results show an increased percentage of cells in the G2 phase in comparison with the control in Molt-4 (Figure 4(d)) and CEM (Supplementary 3(a)) treated cells. Furthermore, there is a low percentage of DNA degradation of 17% in Mol-4 (Figure 4(e)) and CEM (Supplementary 3(b)) cells treated with I-CRP at 24 hours of treatment. These results demonstrate that I-CRP induces DNA damage (*γ*-H2AX), leading to p53 augmentation, and cell cycle arrest in the G2 phase, suggesting a classical apoptosis cell death in T-ALL cell lines.

### 3.5. IMMUNEPOTENT-CRP Induces Caspase-Dependent Cell Death in T-ALL Cells

**Figure 5:**
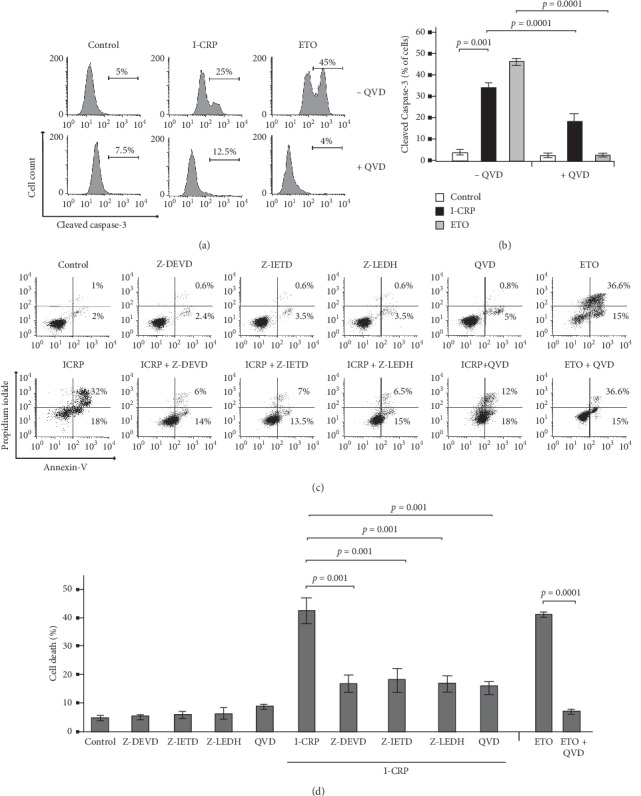
Caspase-3 activity and effects of caspase inhibition on I-CRP-treated Molt-4 cells. (a) Representative histogram of caspase-3 activity analysis and (b) quantification by flow cytometry with FITC-DEVD-FMK staining in Molt-4 cells treated with after I-CRP and etoposide (ETO), using QVD as a pan-caspase inhibitor. (c) Representative dot plots of cell death analysis and (d) quantification by flow cytometry with annexin-V and propidium iodide (PI) staining in Molt-4 cells after I-CRP and etoposide (ETO) treatments, using Z-DEVD (caspase-3 inhibitor), Z-IETD (caspase-8 inhibitor), Z-LEHD (caspase-9 inhibitor), and QVD (pan-caspase inhibitor). The results are presented as mean ± standard deviation of three different experiments.

Caspase-3 is the principal caspase effector in apoptosis [6, 27], and previous studies described that I-CRP induces cleavage of capase-3 but triggers caspase-independent cell death mechanism in tumor cells [21]. To determine the cell death mechanism induced by I-CRP in Molt-4 cells, we tested caspase-3 activation induced by I-CRP and etoposide (ETO) as an apoptotic inductor (positive apoptosis inductor control) in presence or absence of QVD-OPh (QVD; as a pan-caspase inhibitor). In Molt-4 cells, I-CRP induced slight caspase-3 activation from 5% to 25% (Figure 5) at 24 hours of treatment. Further, QVD prevented caspase-3 activation induced by I-CRP and ETO (Figure 5(b)). To reveal whether I-CRP induced apoptosis, we used the pan-caspase inhibitor (QVD) and specific caspase inhibitors Z-DEVD (caspase-3 inhibitor), Z-IETD (caspase-8 inhibitor), and Z-LEHD (caspase-9 inhibitor). In Figure 5(c), we can observe that the percentage of cell death diminishes in comparison with the CC_50_ of I-CRP when using caspase inhibitors. Caspase dependence for cell death was confirmed in CEM cells (Supplementary 4(a)), where we also observed cell death inhibition when inhibiting caspases. This indicates that the mechanism of cell death induced by I-CRP is caspase-dependent in T-ALL cells.

### 3.6. Role of Caspases in the Mitochondrial Alterations Induced by I-CRP in T-ALL Cells

**Figure 6:**
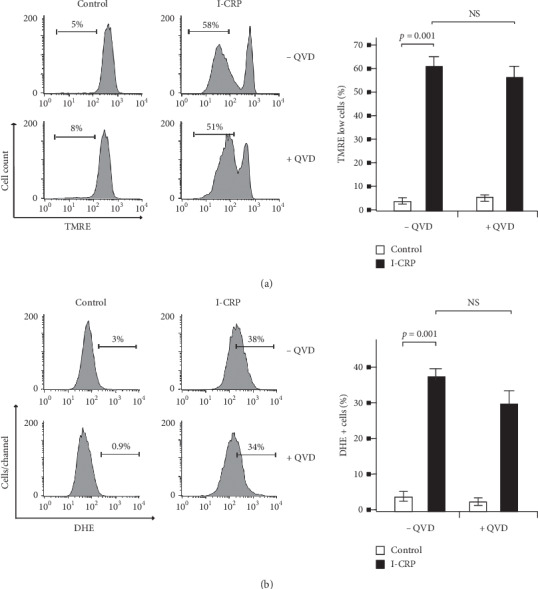
Effect of caspases in mitochondrial damage in Molt-4 cells treated with I-CRP. (a) Representative histogram of mitochondrial membrane potential loss analysis and quantification by flow cytometry using TMRE staining and QVD as a pan-caspase inhibitor. (b) Representative histogram of ROS production analysis and quantification by flow cytometry using DHE staining and QVD as a pan-caspase inhibitor in Molt-4 cells treated with I-CRP. The results are presented as mean ± standard deviation of three different experiments.

The critical step for intrinsic apoptosis is the irreversible and widespread permeabilization of the mitochondrial outer membrane (MOMP), which directly promotes the cytosolic release of apoptogenic factors that induce caspase activation. To analyze the role of caspases in mitochondrial damage, we tested LMMP with TMRE marker and ROS production using DHE stain during caspase inhibition with QVD. We confirmed that I-CRP increased LMMP from 5% to 58%, and that during caspase inhibition with QVD, the LMMP is not changed (51%) (Figure 6(a)); these results were also observed in CEM cells (Supplementary 4(b)). We also observed that during caspase inhibition with QVD, the ROS production is not changed in Molt-4 (34%) (Figure 6(b)) and CEM cells (Supplementary 4(c)). All results suggest that I-CRP induces LMMP and enhances ROS production, which leads to caspase activation and intrinsic apoptosis in T-ALL cells.

### 3.7. IMMUNEPOTENT-CRP Induces ROS-Dependent Cell Death in Leukemic Cells

**Figure 7:**
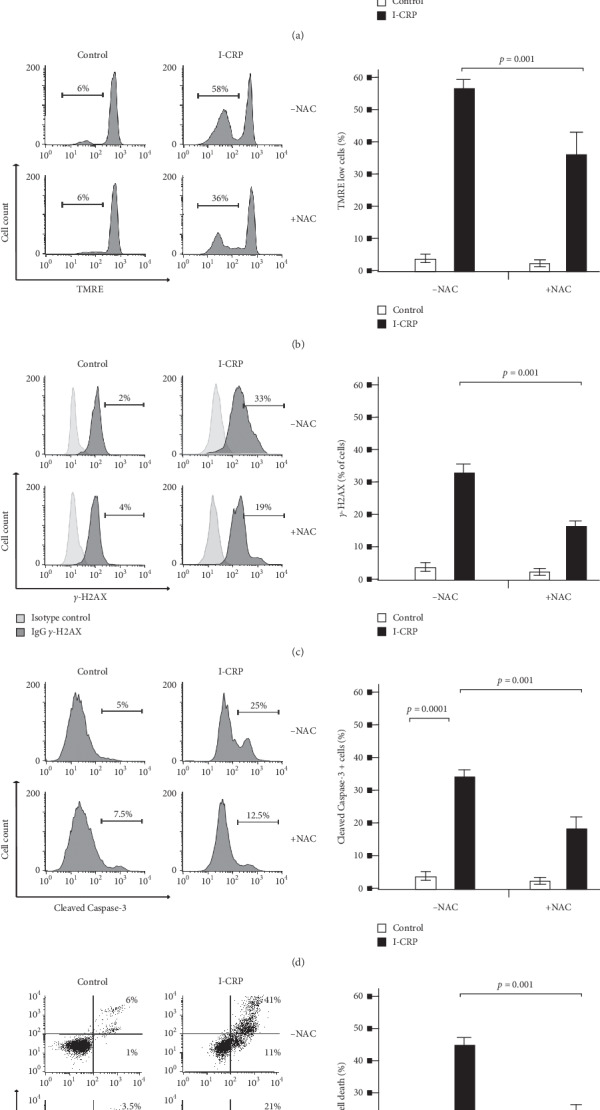
ROS implication in biochemical characteristics of cell death in Molt-4 cells upon I-CRP treatment. (a) Representative histograms of ROS analysis and quantification by flow cytometry using DHE stain and N-Acetyl-cysteine (NAC) as an ROS inhibitor. (b) Representative histograms of mitochondrial membrane potential loss analysis and quantification by flow cytometry using TMRE staining and NAC (ROS inhibitor). (c) Representative histograms of nuclear damage analysis and quantification measure *γ*-H2AX and NAC as an ROS inhibitor by flow cytometry. (d) Representative histograms of caspase-3 activity analysis and quantification by flow cytometry with FITC-DEVD-FMK staining using NAC as an ROS inhibitor. (e) Representative dot plots of cell death analysis and quantification by flow cytometry using annexin-V and propidium iodide (PI) staining during ROS inhibition with NAC. The results are presented as mean ± standard deviation of three different experiments.

An excessive amount of intracellular ROS can result in damage to proteins and DNA, stimulating cell death pathways such as apoptosis [28]. In tumor cells, I-CRP triggers ROS-dependent cell death [21]. To determine the role of ROS in cell death mechanism, first, we tested ROS production in presence of the antioxidant N-acetyl cysteine (NAC), and we assessed its implication in mitochondrial and nuclear damage, caspase-3 activation, and cell death. In Figure 7(a), results exhibit that NAC can repress the ROS production increased by the CC_50_ of I-CRP (0.6 U/mL), demonstrating that NAC is a potential ROS inhibitor. For this, we assessed LMMP during ROS inhibition; in the analysis, we present that NAC avoids LMMP induced by I-CRP (Figure 7(b)). The *γ*-H2AX analysis shows that DNA damage (*γ*-H2AX) diminishes when we block ROS production (Figure 7(c)). The caspase-3 activation analysis showed that the activation of caspase-3 induced by I-CRP is prevented in presence of NAC (Figure 7(d)). Since ROS production induces the molecular characteristics of cell death, we analyzed whether ROS production was playing any role in the mechanism of cell death. In Figure 7(e), we observed that cell death induced by I-CRP is blocked during ROS inhibition with NAC in Molt-4 cells. This reveals that I-CRP induces ROS-dependent cell death in leukemic cells, and that it is a conserved cell death mechanism of cancer cells. ROS implication in the cell death mechanism was confirmed in CEM cells, where we also observed ROS, LMMP, and cell death inhibition when we used NAC as an ROS inhibitor (Supplementary 5). Interestingly, the results reveal that cell death diminished during ROS and caspase inhibition. These results demonstrated that ROS production promotes apoptosis in T-ALL cell lines.

## 4. Discussion

The development of selective drugs for cancer treatment is based on the potential of compounds to block cell proliferation and/or induce cell death. Similar treatments for leukemia have been difficult to find because this being a cancer of the immune system and it is unclear whether the drugs will distinguish between healthy and cancerous cells [10]. I-CRP shows selective cytotoxicity to solid cancer cells [16, 20, 21, 23], such as MCF-7, BT-474, MDA-MB-453, A-427, Calu-1, U937, and L5178Y-R cell lines [20, 21, 23]. Other types of DLE have also shown antitumor activity in prostate cancer [29] and can be used in combination with chemotherapies [30] and with antibiotics as manumycin in mouse breast cancer (4T1) [31]. Since these cytotoxic analyses were done in solid cancer cell lines, its effect in cancer lymphocytes was unknown especially because there were doubts about its efficacy in leukemia, as it increases lymphocyte number in lung [14] and breast [15] cancer patients. Here, we report for the first time the cell death mechanism of I-CRP or a DLE in leukemic cells.

I-CRP has selective cytotoxicity in the T-ALL cell lines, without affecting PBMCs. Interestingly, in T-ALL cells, I-CRP cytotoxicity is high in comparison with cervical [21] or lung [23] cancer cells, suggesting that toxicity is cell lineage dependent. Of interest, bDLE at high concentrations induce cell death in K562 cells, while at low concentrations, it induced differentiation toward the monocyte/macrophage and megakaryocytic lineages [32]. The explanation could be that I-CRP may induce lymphocyte activation and, as a consequence, cell death, as it occurs in activation-induced cell death in T-cells [33].

I-CRP showed mitochondrial alterations including high ROS production, loss of MMP, and increased Bax (proapoptotic protein) and decreased Bcl-2 (anti-apoptotic protein) expressions. Similar results were found in curcumin-induced apoptosis, where upregulation of the Bax/Bcl2 ratio in SW872 was found [34]. Bcl-2 plays an important role in progression of cancer and resistance to apoptosis and is usually increased in leukemia [34–36]. However, I-CRP prevents apoptotic resistance from leukemic cells. This is similar to resveratrol, prednisone, [37] and prednisolone that induces apoptosis in CCRF-CEM cell lines via Bax/Bcl-2 [37].

Here, we show that I-CRP induces nuclear damage including DNA damage (*γ*-H2Ax), p53 overexpression, cell cycle arrest in the G2 phase, and DNA degradation. This mechanism is similar to daunorubicin-induced cell death in human acute lymphoblastic leukemia (CCRF-CEM and Molt-4) [38] and cell death induced by etoposide in HeLa cells [39], which induce DNA break and overexpression of *γ*-H2Ax [39]. Accordingly, the HeLa cells show cell cycle arrest in the G2 phase and low DNA degradation (less than 20%) in response to exposure to I-CRP at 16 h and 24 h [21], which means that *γ*-H2Ax and p53 overexpression may be also involved in HeLa cells.

The activation of caspases is essential for apoptosis; we tested the caspase-3 activity and detected caspase-3 activation, and during the blocking of caspase-3, caspase -8, and caspase -9, cell death was inhibited, indicating apoptosis in the leukemia cell line. Similarly, in murine lymphoblastic leukemia cells (L1210), three different chalcones (flavonoids) induce stress in the endoplasmic reticulum, leading to intrinsic and extrinsic apoptosis [40]. I-CRP induces caspase-3 activation but caspase-independent cell death mechanisms in cervical cancer cells [21], while cell death is also caspase independent in lung cancer cells [23], suggesting that I-CRP induces different cell death modalities depending on the type of cancer.

ROS play a central role in cell signaling, in regulation of the main pathways of apoptosis mediated by mitochondria, death receptors, and endoplasmic reticulum (ER) [28]. In hematological cells, ROS promote cell survival, activation, migration, and proliferation, but oxidative stress has been observed in several hematopoietic malignancies [41–43]. I-CRP increases ROS production, which is inhibited in presence of NAC; also, NAC inhibits the loss of MMP, DNA damage, caspase-3 activation, and cell death in Molt-4 cells. Similarly, 4-HRP induces ROS-dependent cell death in CCRF-CEM, CRF-HSB2, Molt-4, and KG-1 [44]. In Jurkat [45] and in hDPC [46] cells, ROS inhibition blocked extrinsic apoptosis induced by the FAS receptor. CH-AuNPs induce caspase-3 activation, leading to ROS-dependent apoptosis in HeLa [25] and CCRF-CEM [47] cells.

Our results demonstrate that I-CRP can play different roles in healthy and cancerous lymphocytes. Interestingly, cell death of T-lymphocytes following their activation involves extrinsic apoptosis [33, 48]. For this, it is important to evaluate if I-CRP induces Molt-4 activation and, as a consequence, apoptosis.

## 5. Conclusion

**Figure 8:**
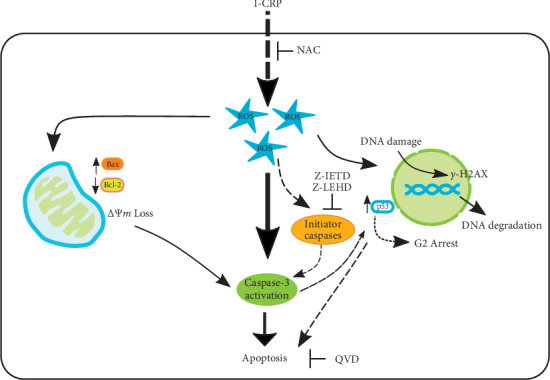
Schematic representation of cell death mechanism in T-ALL cells treated with I-CRP. We propose a cell death mechanism induced by I-CRP in leukemic cells. I-CRP promotes ROS production, which induces DNA damage (*γ*-H2Ax) in the nucleus with overexpression of p53 to induce cell cycle arrest in the G2 phase and finally DNA degradation. Additionally, ROS production induces mitochondrial damage (loss of mitochondrial membrane potential), including pro- and anti-apoptotic protein modulation (Bax and Bcl-2) and finally caspase-3 activation, inducing apoptosis.

Altogether, our results confirm that I-CRP induces selective cytotoxicity in leukemic cells, diminishing cell viability and increasing cell death without affecting healthy blood cell populations. The regulated cell death mechanism induced by I-CRP in nonsolid tumors is different in comparison with solid tumors; however, they share characteristics, such as a cell cycle arrest and production of ROS. Additionally, I-CRP promotes ROS production, which induces DNA damage (*γ*-H2Ax) in the nucleus with overexpression of p53 to induce cell cycle arrest in the G2 phase and finally DNA degradation. ROS production induces mitochondrial damage (loss of mitochondrial membrane potential), including pro- and anti-apoptotic protein modulation (Bax and Bcl-2) and finally caspase-3 activation, inducing apoptosis in T-ALL cells (Figure 8). This work opens the door to evaluate in more detail the importance of cell lineage in the selectivity and the mechanism of cytotoxicity of I-CRP, which might contribute to its widespread use and clinical application.

## Data Availability

The data used to support the findings of this study are available from the corresponding author upon request.

## Supplementary Materials

Supplementary Materials Supplementary figure 1: morphological analysis of T-ALL cell lines after I-CRP treatment. Supplementary figure 2: mitochondrial alterations in CEM cell line and PBMC upon I-CRP treatment. Supplementary figure 3: cell cycle and DNA degradation analysis in the CEM cell line after I-CRP treatment. Supplementary figure 4: caspase implication in cell death mechanism of I-CRP in CEM cells. Supplementary figure 5: ROS implication in cell death mechanism of I-CRP in CEM cells.
