# Supplementary material for: Bovine Dialyzable Leukocyte Extract IMMUNEPOTENT-CRP Induces Selective ROS-Dependent Apoptosis in T-Acute Lymphoblastic Leukemia Cell Lines
Source: J Oncol. 2020 Jun 8;2020:1598503. doi: 10.1155/2020/1598503 (PMC7298273; doi:10.1155/2020/1598503)
Supplement: Supplementary Materials — Supplementary figure 1: morphological analysis of T-ALL cell lines after I-CRP treatment. Supplementary figure 2: mitochondrial alterations in CEM cell line and PBMC upon I-CRP treatment. Supplementary figure 3: cell cycle and DNA degradation analysis in the CEM cell line after I-CRP treatment. Supplementary figure 4: caspase implication in cell death mechanism of I-CRP in CEM cells. Supplementary figure 5: ROS implication in cell death mechanism of I-CRP in CEM cells. [file 1598503.f1.pdf]

Bovine Dialyzable Leukocyte Extract IMMUNEPOTENT-CRP induces selective ROS-dependent apoptosis in T-Acute Lymphoblastic Leukemia cell lines

Helen Yarimet Lorenzo-Anota[1], Ana Carolina Martínez-Torres[1], Daniel Scott-Algara[2], Reyes S. Tamez-Guerra[1], Cristina Rodríguez-Padilla[1]

1. Universidad Autónoma de Nuevo León, Facultad de Ciencias Biologicas, Laboratorio de Inmunología y Virología, Mexico.
2. Unité de Biologie Cellulaire des Lymphocytes, Institut Pasteur, Paris, France.

Corresponding Author:
Ana Carolina Martínez-Torres. Pedro de Alba s/n. Ciudad Universitaria, A.P. 124-F. C.P. 66451, San Nicolás de los Garza, Nuevo León, México T. (+52) 81 83.29.41.15 / 83.76.43.19 / Fax. (+52) 81 83.52.42.12.
ana.martinezto@uanl.edu.mx

Supplementary Material

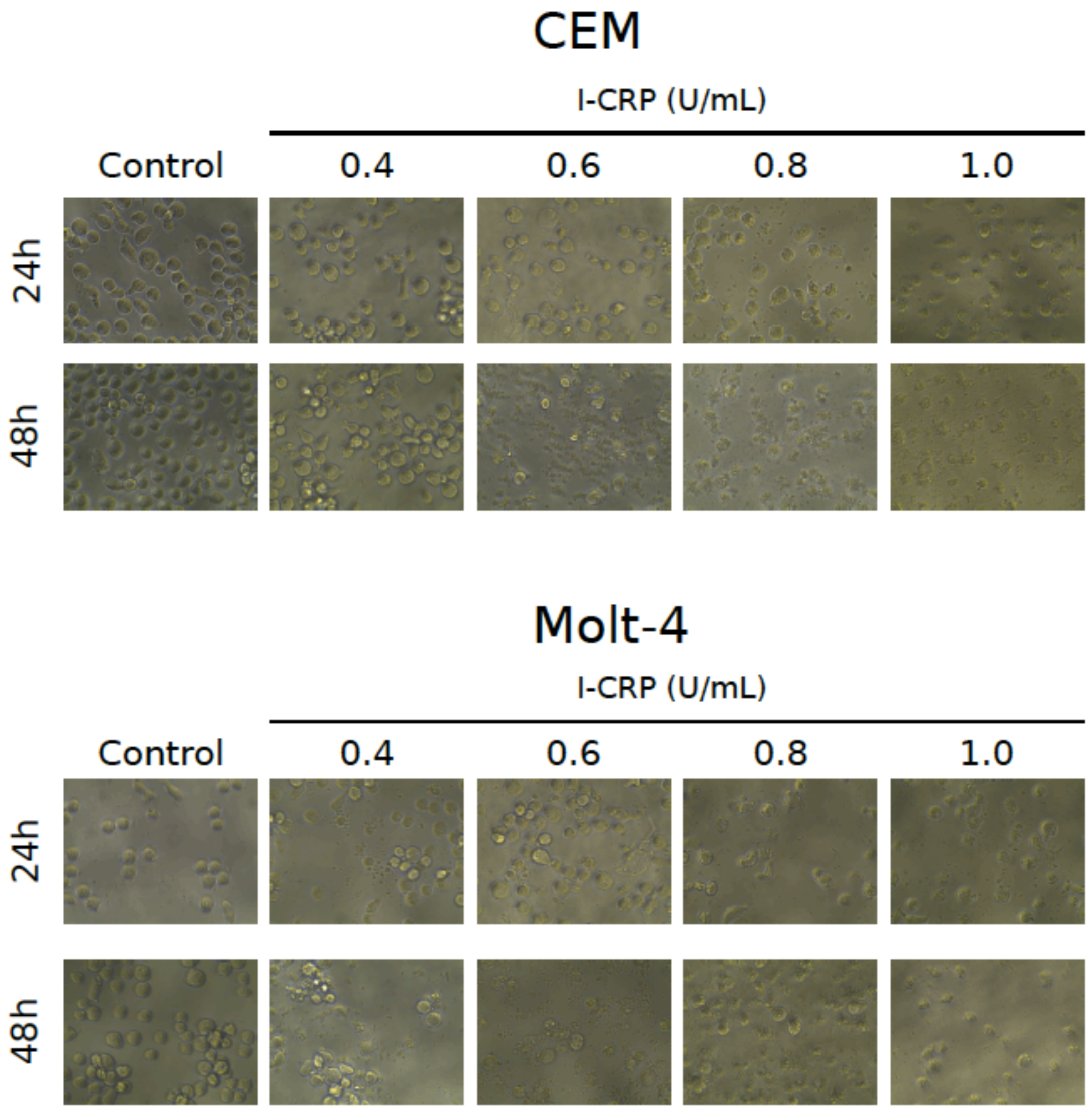

Supplementary 1. Morphological analysis of T-ALL cell lines after I-CRP treatment. Representative images of optical microscopy of CEM and Molt-4 cells treated at different concentrations (0.4, 0.6, 0.8, 1.0U/mL) of I-CRP for 24 and 48 hours.

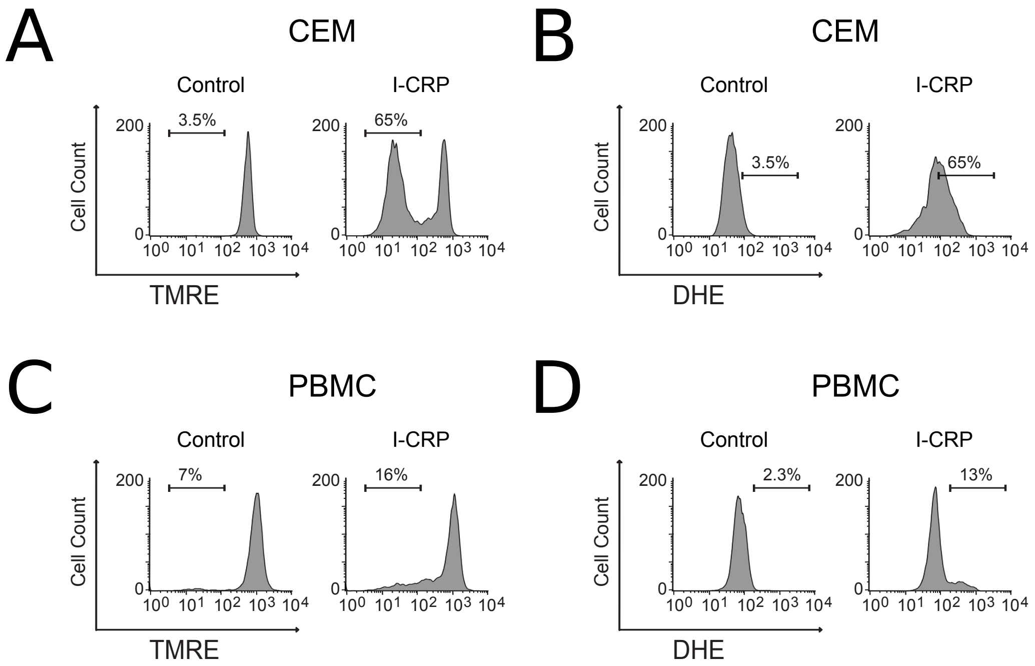

Supplementary 2. Mitochondrial alterations in CEM cell line and PBMC upon I-CRP. **A)** Representative histogram of mitochondrial membrane potential loss analysis, using TMRE by Flow cytometry in CEM cells. **B)** Representative histogram of ROS analysis by Flow cytometry using DHE stain in CEM cell line. **C)** Representative histogram of mitochondrial membrane potential loss analysis, using TMRE by Flow cytometry in PBMC. **D)** Representative histogram of ROS analysis by Flow cytometry using DHE stain in PBMC.

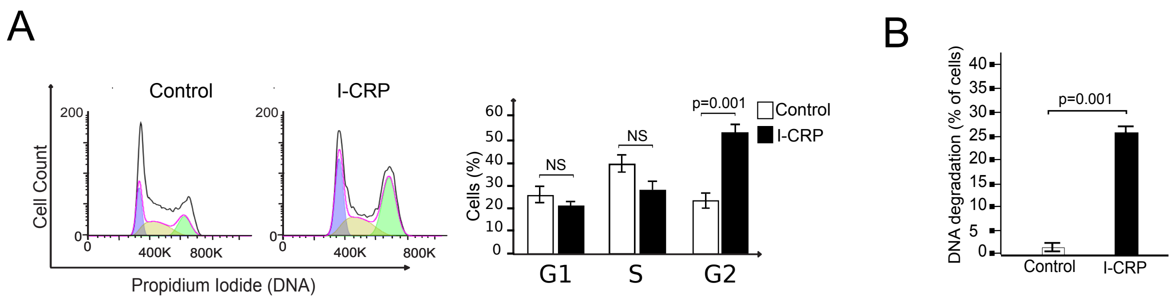

Supplementary 3. Cell cycle and DNA degradation analysis in CEM cell line after I-CRP treatment. A) Representative histogram of cell cycle analysis and quantification, using RNAse and propidium iodide (PI) stain, by Flow cytometry. **B)** DNA degradation quantification by Flow cytometry. The results are presented as mean ± standard deviation of three different experiments.

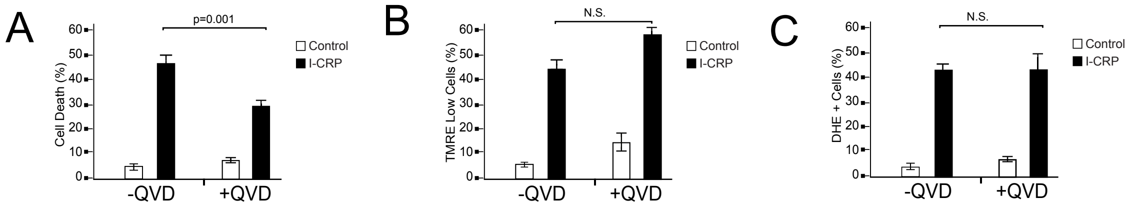

Supplementary 4. Caspases implication in cell death mechanism of I-CRP in CEM cells. A) Graph represents the mean of the cell death quantification by Flow cytometry using Annexin-V and propidium iodide (PI) staining in cells left untreated (control) or treated with ICRP, with or without caspases inhibition with QVD. **B)** Graph represents the mean of the mitochondrial membrane potential loss quantification by Flow cytometry using TMRE staining in cells left untreated (control) or treated with ICRP, with or without QVD as a pan-caspase inhibitor. **C)** Graph represents the mean of the ROS production quantification by Flow cytometry using DHE staining, in cells left untreated (control) or treated with ICRP, with or without QVD as a pan-caspase inhibitor. The results are presented as mean ± standard deviation of three different experiments.

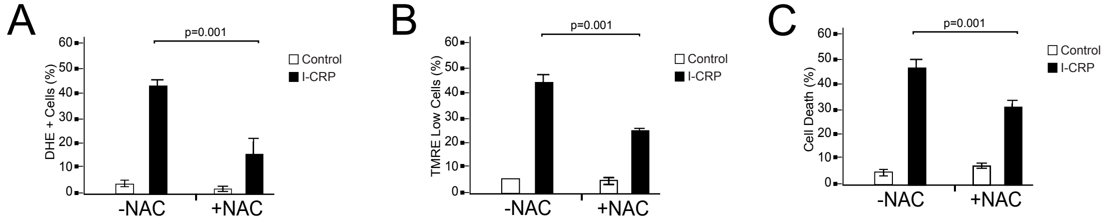

Supplementary 5. ROS implication in cell death mechanism of I-CRP in CEM cells. A) ROS quantification by Flow cytometry using DHE staining in cells left untreated (control) or treated with ICRP, with or without N-Acetyl-cysteine (NAC) as a ROS inhibitor. **B)** Mitochondrial membrane potential loss quantification by Flow cytometry using TMRE staining in cells left untreated (control) or treated with ICRP, with or without NAC (ROS inhibitor). **C)** Cell death quantification by Flow cytometry using Annexin-V and propidium iodide (PI) staining in cells left untreated (control) or treated with ICRP, with or without ROS inhibition with NAC. The results are presented as mean ± standard deviation of three different experiments.
